# Rabies and the pandemic: lessons for One Health

**DOI:** 10.1093/trstmh/trab123

**Published:** 2021-08-14

**Authors:** Deborah Nadal, Sarah Beeching, Sarah Cleaveland, Katy Cronin, Katie Hampson, Rachel Steenson, Bernadette Abela-Ridder

**Affiliations:** Institute of Biodiversity, Animal Health & Comparative Medicine, University of Glasgow, Glasgow, G12 QQ, UK; Oshun Partnership, 19 Cedar Road, Sutton, SM2 5DA, UK; Institute of Biodiversity, Animal Health & Comparative Medicine, University of Glasgow, Glasgow, G12 QQ, UK; Oshun Partnership, 19 Cedar Road, Sutton, SM2 5DA, UK; Institute of Biodiversity, Animal Health & Comparative Medicine, University of Glasgow, Glasgow, G12 QQ, UK; Institute of Biodiversity, Animal Health & Comparative Medicine, University of Glasgow, Glasgow, G12 QQ, UK; Department of Control of Neglected Tropical Diseases, World Health Organization, Geneva 1202, Switzerland

**Keywords:** COVID-19 pandemic, disease preparedness, neglected tropical diseases, One Health, rabies, rabies elimination

## Abstract

This article examines the impact of coronavirus disease 2019 (COVID-19) on dog-mediated rabies, a neglected tropical disease that remains endemic in >65 countries. A globally agreed strategy for rabies elimination is underpinned by a One Health approach, coordinating human and animal health sectors and engaging communities. We present data on the scale and nature of COVID-19 disruption to rabies control programmes and the wider learning for One Health implementation. We argue that the global shift in health priorities caused by the pandemic, and consequent side-lining of animal health, will have broader ramifications for One Health implementation and preparedness for future emergent zoonoses.

## Introduction

Essential health services have been disrupted across the globe by the coronavirus disease 2019 (COVID-19) pandemic, exacerbating inequalities and setting back communities already suffering a high burden of preventable disease. This is especially true for neglected tropical diseases (NTDs) whose control is underpinned by community-level interventions. The launch of the World Health Organization's NTD roadmap renews a commitment to achieving key targets for these diseases by the end of this decade, aligned with the Sustainable Development Goals.^1^

Rabies is among the NTDs prioritised for elimination, with a target of reaching zero human deaths from dog-mediated rabies by 2030.^2^ In 2020, the United Against Rabies Forum was launched to accelerate progress towards this goal, promoting a One Health approach. The key elements of the global rabies strategy are increased mass dog vaccination, improved access to human rabies vaccines and community engagement. Progress on rabies can serve as a tracer for equity of access to health services in underprivileged communities and an early pathfinder for effective One Health implementation.^3^

## Views from the frontline of rabies control

**Figure 1.**
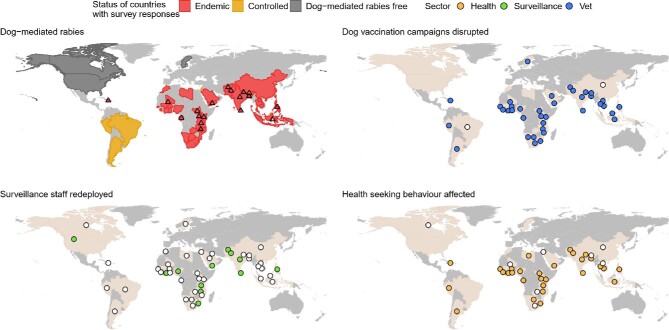
The status of dog-mediated rabies in countries with survey respondents (n=47) and key informant interviews (n=19) and reported disruptions to mass dog vaccination campaigns, redeployment of surveillance staff and effects on health-seeking behaviour of bite victims.

Drawing from the observations of people directly engaged in rabies programmes around the world, we examined the scale and nature of COVID-19 disruptions to rabies control and One Health implementation. We undertook a mixed method online survey, in English, comprising 26 closed-ended and 35 open-ended questions. Participants from government, international organizations, non-governmental organizations (NGOs), industry and academia were invited via the United Against Rabies network. In early 2021 we received 87 completed questionnaires from 47 countries, with four broader international perspectives from the United Nations Children's Fund and the pharmaceutical sector (Figure 1). A subset of 19 participants subsequently participated in an in-depth interview (Figure 1). Summary statistics were calculated from the survey data and qualitative data and interview notes were analysed in Nvivo12 (QSR International, Chadstone, VIC, Australia).

## Impacts on rabies activities

### Human post-exposure prophylaxis

**Figure 2.**
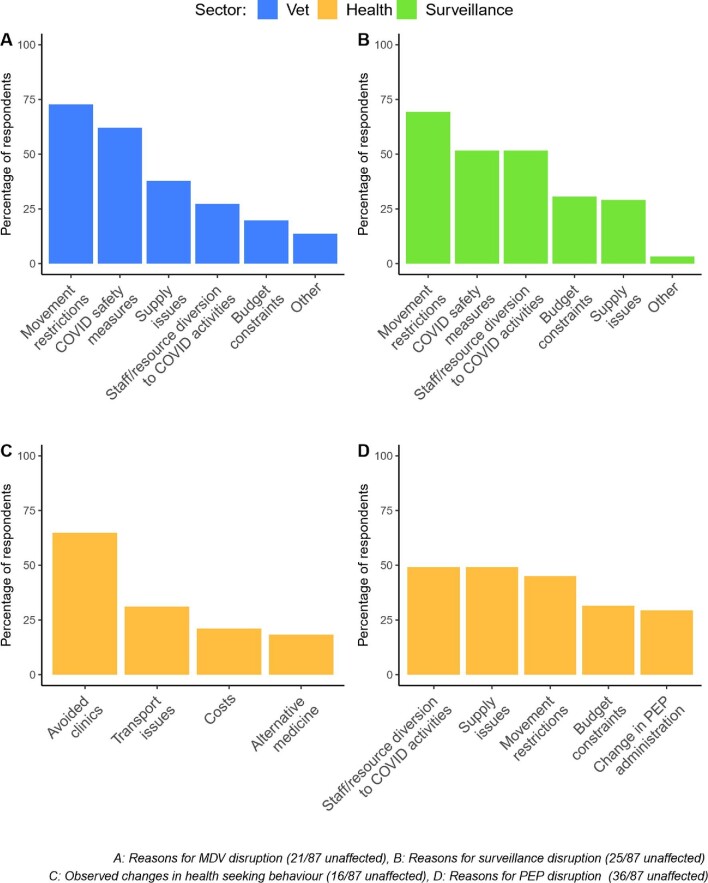
Reasons reported for disruptions to rabies prevention and control in relation to (A) mass dog vaccinations (MDV), (B) surveillance and laboratory diagnosis, (C) health-seeking behaviour of animal bite victims and (D) provisioning of post-exposure prophylaxis (PEP).

Human deaths from rabies are entirely preventable through prompt post-exposure prophylaxis. Across Africa and Asia, structural and financial barriers already limit access to post-exposure vaccines and in 78% of countries, the pandemic worsened access (Figure 1). Multiple reasons underlie this (Figure 2C). According to one respondent, vaccine demand in South Asia declined by 50%. Disrupted provisioning, including closures of dedicated bite treatment centres, proved deadly (Figure 2D). A respondent from the Philippines noted, ‘we had one human case after many years…because the bite centre he went to, which was a big bite centre, was closed and he did not try another’. Even when open, difficulties travelling to urban centres for vaccination were reported. Fear of COVID-19 caused some to avoid big hospitals, ending up in smaller clinics that were closed or without stock and raising challenges for vial-sharing protocols. In Yemen, rabies vaccines that have been available in the private sector despite the war became scarce; elsewhere, people turned to traditional healers.

Several countries reduced human rabies vaccine procurement in 2020 and forecast reduced procurement in 2021 and beyond. Uncertainty is especially acute for countries counting on human vaccines pledged through Gavi's Vaccine Investment Strategy, which remains on hold because of the pandemic. New inequalities have emerged beyond access to vaccines. For example, innovations in telemedicine have enabled remote consultations and home visits, but these mainly benefit the already privileged. Nonetheless, toll-free numbers directing bite victims to the closest open clinics offer promise.

### Dog vaccination

Mass dog vaccination is central to the global strategy for rabies elimination, with a target coverage rate of 70%. In 2020, financial resources allocated to rabies were reduced in 60% of countries and dog vaccination was often the first activity to go, being carried out as planned in just 5% of countries surveyed (Figure 1). Disruptions included delays, prolonged duration and increased costs, while many did not even start (Figure 2A). In northern Uganda, rabies control is notoriously challenging because of chronic underfunding, ‘but nothing has contributed to the escalation of the rabies problem like COVID-19’ and the inflation that followed. Since December 2020, a human fatality and recurrent canine rabies outbreaks have been ascribed to suspended dog vaccinations.

Most endemic countries experienced dog vaccine importation delays, reduced volumes and distribution challenges. As explained by one respondent, this was ‘because initially dog vaccines did not fall under the category of essential goods’. Even where dog vaccines are produced locally, such as in Zambia, budget constraints halted production. Dog owner participation was also severely affected. COVID-19 safety measures restricting movement and face-to-face interactions were compounded by concerns arising from the pandemic. In urban areas, owner participation decreased from fears of COVID-19 exposure, while respondents from Nigeria and South Africa reported concerns of hijackings on deserted roads in rural areas.

Local innovations sought to overcome obstacles, with drive-through vaccinations piloted in affluent urban areas and door-to-door vaccinations added to central point strategies in rural areas. The training of community animal health workers as *in loco* dog vaccinators has accelerated. Others reported that catching urban free-roaming dogs was easier given the reduced traffic. More generally, the use of technologies such as radio advertising of vaccination campaigns and apps for registering dogs has grown.

### Community engagement

Engaging local communities is key to improving health-seeking behaviour and achieving and sustaining high coverage of dog vaccinations. But in >90% of countries, awareness activities in schools and on World Rabies Day were badly affected. When moved online, ‘they can reach many more people, but not those who need them’, such as those with limited education and poor access to the internet. For Mission Rabies, an NGO working in multiple countries, education activities were most affected, mainly because of long-term school closures. In Ghana, 2020 sponsorship for rabies education was withdrawn.

### Outlook

Weak surveillance in rabies-endemic countries makes assessing the pandemic impacts challenging. In 25% of countries, staff who conduct rabies surveillance were reassigned to COVID-19 response (Figure 1) and movement restrictions hampered rabies investigations. Impacts may only become evident belatedly as a consequence of disrupted dog vaccinations and reduced access to post-exposure vaccines, but respondents were clear that globally, ‘momentum that was gaining has been lost’. For countries optimistic about the 2030 goal, hard-won gains have eroded (see Box 1). The consensus is that rabies control activities will not return to pre-COVID-19 levels before 2022, and only if there is high COVID-19 vaccination coverage and rabies is not deprioritized and defunded. Concerns were raised about competing vaccination campaigns or priorities from other zoonotic outbreaks and related food security issues.

Box 1. **The case of Bhutan**

The case of Bhutan, where a rabies death was reported in 2020 for the first time since 2016, illustrates many points. ‘The girl was bitten in the first week of September. The national lockdown had started on 11 August and, in the [border] area where she lived, it was lifted in late September. Her area was both a highly endemic rabies area and a high-risk COVID-19 area. Yet, until then, Bhutan had not a single COVID-19 death. So we definitely need to quickly put some aggressive and extraordinary measures [to address rabies] in place’. This case was attributed, first, to COVID-19-related movement restrictions and the inconvenience of paperwork verifications on the road to the hospital and, second, the cancellation of routine dog ‘vaccination campaigns on the porous southern border’. ‘Because of COVID-19’, the respondent continued, ‘human movements on the border are now strictly controlled, but for dogs, it's very easy to cross it. I hope that now, [global] policymakers realize that rabies should be included in border management activities’.

## Cross-cutting implications

Lessons from tackling rabies during COVID-19 extend beyond this crisis to broader One Health challenges.^4^ Respondents acknowledged the potential for positive outcomes in the longer term, such as improved cold and supply chains, strengthened diagnostic capacity, investment in e-learning and regional coordination. Indeed, recognition of COVID-19 as a zoonosis may increase awareness about the importance of safeguarding animal health as well as the potential for cross-cutting contributions of the veterinary sector, often deployed to COVID-19 contact tracing, laboratory work and vaccination. But despite growing recognition of interdisciplinary One Health approaches, respondents largely believe the pandemic has harmed discussion beyond human health within policy circles.

COVID-19 has highlighted the complexity and challenges of global coordination, national policy and local vaccine delivery. Inequities in vaccine access is a dominant narrative, repeating historical failures of neglected diseases like rabies, where effective vaccines have long existed without reaching those most in need. ‘The essential truth that health is mostly created and destroyed outside of the health sector’^5^ should be a key tenet of the One Health framework. For rabies, surveillance needs expanding beyond clinics and dog vaccination needs mobilizing at rural grassroots, recognizing its reciprocal benefits to human and livestock health. Rabies can significantly contribute to building integrated capacity, taking a whole-government approach, outside institutionalized health structures and silos.

Dog vaccination was reported as the most disrupted rabies control activity, yet it is the most cost-effective action that can be taken. This principle applies well beyond rabies to general One Health approaches, i.e. that animal health interventions often provide cheap and equitable routes to protecting human health. This has been a difficult message to convey at the height of the pandemic, but it is essential to preventing many zoonotic diseases at the source and building emergent disease preparedness into health ecosystems.

Effective management of endemic zoonoses like rabies will build the most robust platform from which to strengthen universal health coverage and global health security. The capacities and activities required for rabies control, from surveillance to vaccinations, building partnerships locally and regionally, with community engagement at the core, directly enable early detection and response to emerging diseases. Investing in One Health through rabies control will save lives, build solidarity, reduce health inequalities and strengthen core capacities for responding to future health emergencies.

## Data Availability

Data available on request from the authors.
